# Alternative Tobacco Product Use in Critically Ill Patients

**DOI:** 10.3390/ijerph17238707

**Published:** 2020-11-24

**Authors:** Tom Liu, Thomas J. Deiss, Matthew W. Lippi, Alejandra Jauregui, Kathryn Vessel, Serena Ke, Annika Belzer, Hanjing Zhuo, Kirsten N. Kangelaris, Antonio D. Gomez, Michael A. Matthay, Kathleen D. Liu, Carolyn S. Calfee

**Affiliations:** 1 Department of Medicine, Division of Pulmonary and Critical Care Medicine, University of California, San Francisco, CA 94143, USA; thomasjdeiss@gmail.com (T.J.D.); mattlippi@gmail.com (M.W.L.); alejandra.jauregui@ucsf.edu (A.J.); kathrynvessel@gmail.com (K.V.); serenak028@gmail.com (S.K.); annikagbelzer@gmail.com (A.B.); hanjing.zhuo@ucsf.edu (H.Z.); antonio.gomez@ucsf.edu (A.D.G.); michael.matthay@ucsf.edu (M.A.M.); kathleen.liu@ucsf.edu (K.D.L.); carolyn.calfee@ucsf.edu (C.S.C.); 2 Cardiovascular Research Institute, University of California, San Francisco, CA 94143, USA; 3 Virginia Tech Carilion School of Medicine, Roanoke, VA 24014, USA; 4 Rutgers—New Jersey Medical School, East Orange, NJ 07103, USA; 5 Center for Tobacco Control Research and Education, University of California, San Francisco, CA 94143, USA; 6 Department of Medicine, Division of Hospital Medicine, University of California, San Francisco, CA 94143, USA; kirsten.kangelaris@ucsf.edu; 7 Department of Medicine, Division of Pulmonary and Critical Care Medicine, Zuckerberg San Francisco General Hospital and Trauma Center, San Francisco, CA 94143, USA; 8 Department of Anesthesia, University of California, San Francisco, CA 94143, USA; 9 Department of Medicine, Division of Nephrology, University of California, San Francisco, CA 94143, USA

**Keywords:** non-cigarette tobacco products, electronic nicotine delivery devices, smoking-caused disease, surveillance and monitoring, alternative tobacco products, critical illness, survey research

## Abstract

*Background:* Alternative tobacco product (ATP) use has bee linked to critical illness, however, few studies have examined the use of these substances in critically ill populations. We sought to examine ATP use within critically ill patients and to define barriers in accurately assessing use within this population. *Methods:* We prospectively studied 533 consecutive patients from the Early Assessment of Renal and Lung Injury study, enrolled between 2013 and 2016 at a tertiary referral center and a safety-net hospital. ATP use information (electronic cigarettes, cigars, pipes, hookahs/waterpipes, and snus/chewing tobacco) was obtained from the patient or surrogate using a detailed survey. Reasons for non-completion of the survey were recorded, and differences between survey responders vs. non-responders, self- vs. surrogate responders, and ATP users vs. non-users were explored. *Results:* Overall, 80% (*n* = 425) of subjects (56% male) completed a tobacco product use survey. Of these, 12.2% (*n* = 52) reported current ATP use, while 5.6% reported using multiple ATP products. When restricted to subjects who were self-responders, 17% reported ATP use, while 10% reported current cigarette smoking alone. The mean age of ATP users was 57 ± 17 years. Those who did not complete a survey were sicker and more likely to have died during admission. Subjects who completed the survey as self-responders reported higher levels of ATP use than ones with surrogate responders (*p* < 0.0001). *Conclusion:* ATP use is common among critically ill patients despite them being generally older than traditional users. Survey self-responders were more likely than surrogate responders to report use. These findings highlight the importance of improving our current methods of surveillance of ATP use in older adults in the outpatient setting.

## 1. Introduction

Alternative tobacco products (ATPs), also referred to as non-cigarette tobacco products, are a broad category of substances that include electronic cigarettes, cigars, pipes, hookahs/waterpipes, and snus/chewing tobacco [1,2]. The prevalence of ATP usage has steadily increased over time; however, the majority of present investigations focus on the surveillance of youth and young adults or on long-term analyses of healthy outpatients [3,4]. Exposure to ATPs is not benign and may be linked to critical illness, as demonstrated by the 2019 cluster of electronic-cigarette-related severe lung injury diagnoses in the United States linked to Vitamin E acetate- and Tetrahydrocannabinol-containing vaping products [5].

Despite this fact, there is little current data on ATP use in critically ill patients (people who are so sick that they require admission to the intensive care unit). It is unknown whether ATP use is a significant issue within critically ill patients, and long-term data on ATP-related harms are only beginning to be investigated [6,7]. It is important to note that critically ill patients are generally older and sicker than the United States general population, with an average age of 60+ [8]. Because adult substance users have a higher prevalence of chronic disease, they may suffer greater harm from continued ATP use, in other words, they may experience greater negative health effects than younger and healthier people [9]. Critical illness is broad, and ATP use may cause harm in multiple ways, including directly causing illness, exacerbating underlying conditions, and amplifying harms caused by unrelated illnesses in patients requiring critical care. Different ATPs may have different health implications, especially since this broad category of substances includes combustible tobacco, non-combustible tobacco, and nicotine-containing products, which carry different risk profiles [10,11,12,13,14]. However, given the potential negative impact of any ATP exposure on health outcomes and the paucity of data on ATP usage in critically ill patients, it is important to identify whether this group warrants further surveillance or intervention [15].

The use of tobacco products in the general population has been investigated through various surveillance systems, in the general population such as the National Health and Nutrition Examination Survey (NHANES), National Health Interview Survey (NHIS), in longitudinal studies such as the Population Assessment of Tobacco and Health (PATH), and in youth populations such as with the National Youth Tobacco Survey (NYTS) [16,17]. In recent years, national surveys such as NHANES and PATH have added questions about electronic cigarette use, showing anywhere between 2% and 5% prevalence in the 40+ age group [18,19,20]. State-level data in the Behavioral Risk Factor Surveillance System (BRFSS) indicate that in adults, smokeless tobacco use ranges from 1% to 10% and electronic cigarette use ranges from 2% to 7% [21]. The National Adult Tobacco Survey (NATS) found that the current prevalence of waterpipe use is about 2% [22]. Furthermore, a few studies have investigated the prevalence of specific ATPs in older adult populations [18]. For example, focused cohorts of non-hospitalized adults such as data from the Genetic Epidemiology of COPD (COPDGene) and Subpopulations and Intermediate Outcome Measures in COPD Study (SPIROMICS) suggest that certain ATPs are prevalent in adult populations who have chronic diseases [23,24]. However, there are no studies on cohorts of adults who are experiencing critical illness. Moreover, general populations do not include hospitalized patients and may have response biases based on age, gender, or a variety of other factors [25]. Since ATP exposure may cause critical illness or exacerbate underlying conditions, it is important to begin creating systems that will allow researchers and clinicians to measure the effects of ATP exposure on critical illness.

Unfortunately, identifying substance use can be difficult once patients are ill because patients may be sedated or have altered mental status, because patients are too ill to participate, or because patients die before consent can be obtained for research purposes. The challenges are especially relevant within the high-acuity setting of critical care and pose a barrier to both patient enrollment and survey data collection [26,27,28,29]. This study was designed to measure ATP use in critically ill patients using a diverse cohort of acutely ill patients who were enrolled as part of a prospective study on the effects of cigarette smoking and acute lung injury. In addition, we also aimed to identify barriers that prevented accurate assessment of ATP use within this cohort.

## 2. Methods

### 2.1. Sample

Participants included in analysis are 533 patients enrolled between October 2013 and December 2016 in the Early Assessment of Renal and Lung Injury (EARLI) study, a prospective cohort of patients admitted directly from the emergency department (ED) to the medical/surgical intensive care units (ICUs)at a tertiary care center (Hospital A), and a safety-net hospital (Hospital B). The majority of included patients (*n* = 414) were identified when an admission request to the ICU was placed from the emergency department. A subgroup of patients also included in this cohort were (*n* = 119) admitted directly to the hospital floor after triggering a sepsis alert in the emergency department. Thus, all participants in this study were patients who were admitted to the hospital for acute illness between October 2013 and December 2016.

### 2.2. Enrollment of Patients

All ED-to-ICU admission alerts and ED-sepsis alerts were transmitted to research staff via an alpha-numeric paging system linked to the EPIC electronic medical record at the time of the admission request. Following a brief screening of the medical record to exclude patients who had a primary neurological diagnosis, potential patients were approached for study enrollment.

All patients (*n* = 267) or their surrogates (*n* = 179) provided informed consent, with the exception of (1) patients who died before they or their surrogate could be approached for informed consent (*n* = 79), or (2) patients whose critical illness prevented them from providing consent and a surrogate could not be identified after 28 days (*n* = 8). These patients were included under an IRB-approved waiver of consent for minimal-risk research based on the FDA guideline 45 Code of Federal Regulations 46.116(f). Informed consent was obtained from the patient by research staff. However, if the patient was unable to provide consent because their critical illness caused them to be incapacitated (sedated or have altered mental status, intubated, or otherwise too ill to participate) consent was obtained from a surrogate. These patients did not have capacity to consent for participation in the study by themselves, and thus, a legally authorized representative (surrogate) was identified [30]. Patients were excluded if they were admitted for primary neurological or neurosurgical diagnosis without significant medical comorbidities.

A total of *n* = 678 participants were determined eligible for enrollment in this study based on inclusion criteria. After accounting for patient refusal (*n* = 97), exclusion because of inability to obtain consent (*n* = 47), or patient withdrawal from study (*n* = 1), *n* = 533 patients were included for our analysis. The 47 patients for whom we were not able to obtain consent most commonly occurred in the situation where the patient may have been capable of providing consent or a suitable surrogate was identified, however due to social factors associated with their critical illness, the research staff did not approach the patient or their family so as to limit undue stress. The patient may have subsequently remained permanently altered or recovered, but informed consent was not obtained and thus these patients were excluded from study. Information about the enrollment of this cohort has also been published previously [31,32,33]. Compensation was not provided for participation in this study. The Institutional Review Board of the University of California, San Francisco, approved the protocol for the enrollment of these patients (Code: 10-02852).

### 2.3. Collection of Data

Clinical characteristics including past medical history, demographics, and baseline vital information from the first 24 h of ICU admission were collected by review of the electronic health record at each hospital by trained research staff. Because of the diverse population of our dataset, including the possibility of data variation due to severity of illness and respondent type, we evaluated clinical characteristics by hospital, survey respondent vs. non-respondent, and self- vs. surrogate respondents. We also evaluated reasons for non-completion of the survey in enrolled patients.

Current ATP usage information (including smokeless tobacco, cigars, little cigars, pipe, hookah, and electronic cigarettes) was obtained from either patient or surrogate as a part of a standardized verbal survey administered by trained research staff at the time of enrollment based on usage within the 30 days prior to admission (Supplemental Figure S1). Respondents who reported use in the 30 days prior to admission, independent of frequency, were defined as any-ATP. In addition, the Alcohol Use Disorders Identification Test (AUDIT) was also given at the same time and used to define alcohol use and abuse [34]. In patients who were initially enrolled under the waiver of consent for whom informed consent was later obtained, verbal survey was conducted at the time of informed consent (within 28 days of enrollment). Thus, patients for whom informed consent was not obtained because of their illness or lack of a surrogate did not complete a survey.

Usage of ATPs was compared to use of cigarettes. Cigarette smoking history was obtained through both verbal survey and chart review. For our survey, patients were asked whether they had smoked more than 100 cigarettes in their lifetime. If they responded “yes,” “Current smoking” was defined as patients who were actively smoking 1 or more cigarettes a day, and “Former smoking” was defined as patients who had quit smoking. “Ever smoking” by survey definition were patients who were current or former smokers [4,35]. Smoking history defined by chart review was gathered from the social history section of the history and physical note from the same admission as the study, or from previous admissions notes if not readily available. ATP-cigarette use was defined as current smoking based on chart review and use of any ATP product. Agreement and inter-rater reliability assessed between the electronic medical record and patient or surrogate response was used to examine the extent to which the medical record and our survey response were measuring similar responses, measuring the reliability of our verbal survey [36,37]. Thus, it was a rough proxy of the accuracy of our survey. Alcohol abuse was defined as an AUDIT score greater than or equal to 8 [34].

Additional clinical characteristics included baseline comorbidity categories obtained from the medical record: coronary artery disease (CAD), congestive heart failure (CHF), hypertension, peripheral vascular disease, cerebrovascular disease, chronic obstructive pulmonary disease (COPD), reactive lung disease, reactive airway disease, home oxygen, and solid tumor with or without metastasis. The comorbidities were grouped as chronic heart disease (CAD or CHF), chronic lung disease (COPD, restrictive lung disease, or supplemental home oxygen), cerebrovascular disease, or solid tumor malignancy.

Patients were followed for 60 days in order to determine mortality status after admission to the hospital. In-hospital mortality was defined as death prior to discharge. If a patient continued to be hospitalized at 60 days, but was alive at 60 days, they were considered to be “alive.”

The severity of illness at the time of ICU admission may influence the ability of enrolled patients to respond to survey questions and was calculated for each patient using the Acute Physiology, Age, Chronic Health Evaluation (APACHE III) prognostic score, which is a set of equations that uses data collected from the first 24 h of ICU admission to predict mortality risk [38]. The APACHE III score consists of several variables including age, sex, race, preexisting comorbidities, several physiological variables, and even location prior to ICU admission. Each five-point increase in APACHE III score (0–299) is independently associated with an increase in the relative risk of hospital mortality. To clarify, the calculation of the APACHE III score is based on a number of physiologic variables and illustrates the risk of mortality in critically ill patients. This is distinct from the number of comorbidities or chronic illnesses that an individual has.

### 2.4. Statistical Analysis

Categorical data were analyzed by Pearson’s chi-squared test or Fisher exact test. Continuous variables were compared using Student’s t-test. For non-normally distributed values, Mann–Whitney U test was used. Statistical significance was defined as *p* ≤ 0.05, using a two-tailed test of hypothesis. Agreement between chart and survey was determined by measuring percent agreement and calculating Cohen’s kappa, a statistical measure of inter-rater reliability [36]. All analyses were performed with R 3.2.0 and validated in STATA (StataCorp. 2011. Stata Statistical Software: Release 12. College Station, TX: StataCorp LP). Inter-rater reliability was analyzed using the IRR package in R 3.2.0.

## 3. Results

### 3.1. Cohort Description

Of the 533 enrolled participants, 425 participants or their surrogates completed a tobacco product survey, an 80% response rate (Supplemental Figure S2). Of the 425 participants with a complete survey, 259 (61%) completed the survey themselves, whereas 166 (39%) had a surrogate respond for them. Reasons for non-completion of the survey included death prior to survey completion (*n* = 60), discharge prior to survey (*n* = 4), refusal to complete survey (*n* = 6), and other/unknown (*n* = 38).

### 3.2. Baseline Characteristics of Study Participants

The baseline characteristics of the study cohort (*n* = 533), stratified by enrollment location, are described in Table 1. Overall, there were no statistically significant differences between Hospital B and Hospital A in survey completion rates (84% vs. 79%; *p* = 0.37), age, or sex. Patients at Hospital B were more likely to be African American (24% vs. 13% at Hospital A, *p* = 0.002) or Hispanic (Latinx) (22% vs. 11%, *p* = 0.01). Cigarette smoking assessed by both survey (*p* = 0.002) and chart review (*p* = 0.004) was more prevalent at Hospital B, however use of 2+ ATPs was similar between the two sites (10% vs. 14%; Hospital A vs. Hospital B; *p* = 0.30).

### 3.3. Clinical Characteristics of Survey Responders vs. Non-Responders

We further compared the clinical characteristics of survey responders to those of non-responders (Supplemental Table S1). Survey responders were younger (65 ± 16 years vs. 69 ± 15, *p* = 0.01), and were more likely to have been admitted to the hospital ward (26% vs. 9%, *p* < 0.0001). There were no statistically significant differences in enrollment hospital, sex, race, or ethnicity between survey responders and non-responders. Survey non-responders were more severely ill (APACHE III: 116 ± 46 vs. 76 ± 35, *p* < 0.0001) and were more likely to have died during the admission in which they were enrolled to the study (71% vs. 14%, *p* < 0.0001).

### 3.4. Clinical Characteristics of Self- vs. Surrogate Responders

We also compared characteristics of self- and surrogate survey responders for the cohort because of the differential responses of ATP use within the two groups (Supplemental Table S2). Cigarette smoking history was obtained from both survey data and charts to measure correspondence between the two. Self-response accounted for 61% (*n* = 259) of total tobacco product use surveys. Notably, patients who completed the survey as self-responders were significantly younger (61 ± 15 vs. 70 ± 15, *p* < 0.0001) and more likely to be Caucasian (*p* = 0.0001) than patients who had a surrogate responder complete the survey for them. In addition, self-responders were more likely to be current cigarette smokers by chart review (*p* = 0.001). Unsurprisingly, self-responders were more likely to have been admitted to the hospital floor (30% vs. 22%), although this was not statistically significant (*p* = 0.051). We used pre-admission residency in a skilled nursing facility as a crude measurement of baseline functional status, which may have affected the ability to complete a survey; however, there were no statistically significant differences between the two groups (Supplemental Table S2). In addition, there were no statistically significant differences in enrollment location, ethnicity, or admission location. These findings persisted in a sensitivity analysis excluding floor patients.

Cigarette usage history reported by survey response was compared to history obtained from chart review (Supplemental Table S3). Self and surrogate responses demonstrated 86.1% and 85.6% agreement with chart review, respectively. Adjusting for chance agreement, both surrogate and self-responders demonstrated moderately strong agreement with the electronic record (Cohen’s κ = 0.75, 0.81).

Finally, among self-responders, we compared characteristics of ATP users vs. non-ATP users (Table 2). ATP users were more likely to be male (82% vs. 59%, *p* = 0.01) and more likely to be current cigarette smokers as measured by survey (*p* < 0.0001) and chart review (*p* < 0.0001). Overall, ATP users reported more alcohol use (51% vs. 27%, *p* = 0.001) and misuse (16% vs. 5%, *p* = 0.008). There were no statistically significant differences in enrollment location, level of care, age, race–ethnicity, or insurance status. Mortality and severity of illness did not differ among ATP users in floor or ICU subsets of patients. ATP users and non-ATP users did not differ with respect to rates of chronic heart disease, history of stroke, chronic lung disease or malignancy (data not shown). Characteristics of ATP users were also compared to characteristics of “only smokers” (Supplemental Table S4).

### 3.5. Prevalence of Alternative Tobacco Product Usage

**Figure 1:**
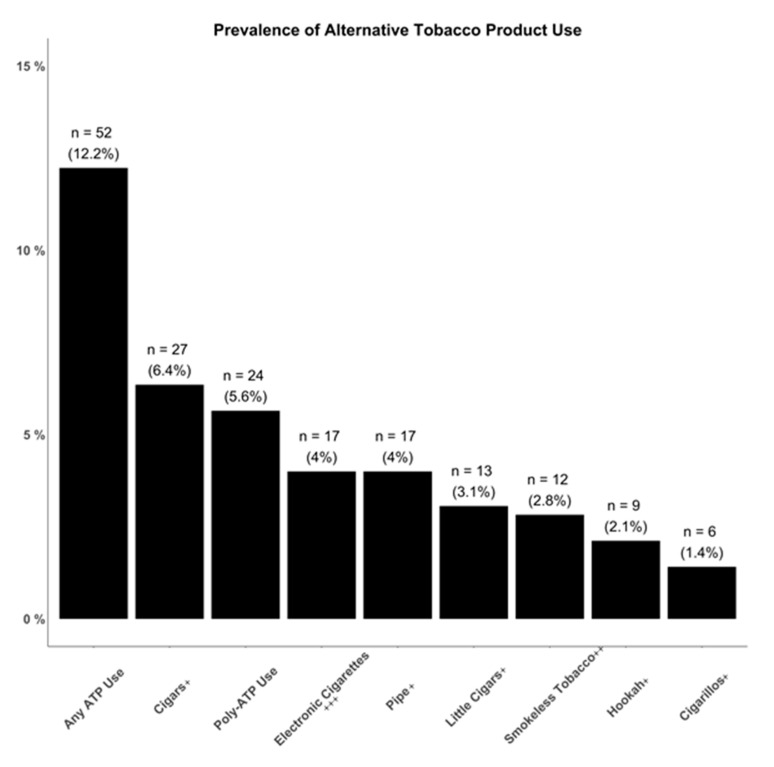
Prevalence of alternative tobacco product use. It is important to note that there may be overlap in the groups displayed, as an individual who reported using both electronic cigarettes and pipes would be counted in the graph for three groups: poly-ATP use, electronic cigarettes, and pipe. + Combustible tobacco product; ++ non-combustible tobacco product; +++ may contain nicotine or other products.

Of the participants with a completed survey, 12.2% (*n* = 52) reported use of any ATPs, while 5.6% (*n* = 24) reported currently using multiple ATP products (Figure 1). Overall, 4% (*n* = 17) reported using electronic cigarettes, 2.8% (*n* = 12) reported using smokeless tobacco, 6.4% (*n* = 27) reported using cigars, 3.1% (*n* = 13) reported using little cigars, 1.4% (*n* = 6) reported using cigarillos, 4% (*n* = 17) reported using pipe, and 2.1% (*n* = 9) reported using hookah (Figure 1).

Analysis of survey responses demonstrated a difference in reported ATP usage between self- and surrogate responders (Table 3). Overall, ATP use was more frequently reported by self-responders (17% vs. 5%, *p* < 0.0001). This difference was observed in cigars (9% vs. 2%, *p* = 0.007), pipe (6% vs. 1%, *p* = 0.02), and ATP-cigarette (any ATP AND cigarette use) (8% vs. 1%, *p* = 0.005) users. Rates of smokeless tobacco (4% vs. 1%, *p* = 0.14), electronic cigarettes (5% vs. 2%, *p* = 0.21), and little cigar (4% vs. 1%, *p* = 0.09) use were not statistically significant.

### 3.6. Barriers to Collection of ATP Data in Critically Ill Patients

We reviewed records of enrollment that were kept by our trained research staff during the study period. Common, patient-related issues that our research staff encountered during enrollment included difficulty in contacting surrogates, language barrier, refusal to participate because they were already participating in too many research studies, and refusal to complete the survey because the patient felt too weak. Common logistical issues included being unable to enroll or administer surveys to patients who needed frequent procedures and being discharged before the survey could be administered. Overall, of 678 patients who were eligible for inclusion, 7% (*n* = 47) of patients or their family members refused participation, 14% (*n* = 97) of patients were excluded because a suitable surrogate was identified but unable to be contacted or capacity to consent was never regained following their illness. One patient later withdrew consent for the study and the information collected was discarded.

## 4. Discussion

To our knowledge, this study is the first to report the prevalence of ATP use in a multicenter cohort of acutely and critically ill patients—a unique population that is often under-studied because of the complexity and cost of data collection [39]. The prevalence of current ATP use in our study is approximately 12%; however, it was higher within self-responders (17%), which may more accurately reflect ATP use overall. Although accurately measuring ATP use is challenging, our findings suggest that critically ill patients may have a high exposure rate to these potentially disease exacerbating products.

Our findings show that ATP use within critically ill patients is quite common and represents a population of older and sicker adults who may not be commonly perceived to use these substances. Interestingly, the prevalence of alternative product use of adults in the EARLI cohort, despite being older and sicker, is similar to published findings in adults from PATH, NHIS, and Tobacco Products and Risk Perceptions Survey datasets [19,40,41]. Reported electronic cigarette, smokeless tobacco, cigar, and hookah usage did not statistically differ from national trends in previously published findings from wave 1 of the Population Assessment of Tobacco and Health (PATH) dataset obtained in 2014 for the adult age group (Table 4). However, pipe usage was significantly more common (pipe: 4.0% vs. 1.0%, *p* < 0.0001) and cigarillo usage was significantly less common (1.4% vs. 3.4%, *p* = 0.02) in this sample as compared to the PATH data. In addition, reported hookah and electronic cigarette usage did not differ from published trends from the cross-sectional Tobacco Products and Risk Perceptions Surveys and 2014 National Health Interview Survey (NHIS), respectively (Table 4). It is important to note that the patients in the EARLI cohort are on average older than adults in the national datasets. It remains unknown what specific risk factors and attributes may increase risk for critical illness in ATP use. The fact that more granular data are not available limits our ability to draw conclusions about any specific link between ATP use and critical illness but is an important goal of future study.

There remain questions about the true prevalence of ATP use in critically ill patients. While prevalence likely varies by adult population, our finding that 17% of self-responders reported ATP use compared to only 5% of surrogate-responders may indicate that family members are unreliable reporters of ATP use. Additionally, it is possible that patients underreport their use because of misconceptions about the risks of ATP use or concerns of stigmatization [42]. In our study, approximately 40% of survey responses came from a close family member, who may simply not know about a patient’s ATP usage. However, the discrepancies between self- and surrogate report of ATP use may also reflect differences between the two groups. For example, self-responders were younger, more likely to be male, and were more likely to be current cigarette smokers, factors that have been associated with a greater likelihood of ATP usage in other cohorts [4,17,20,43]. Moreover, it is possible that our findings reflect the true prevalence of ATP use within these two groups because the sickest patients (who more often have surrogates respond to the survey) do not use ATPs. Nonetheless, future studies validating the accuracy of patient and surrogate responses with regard to ATP use will improve the authenticity of findings.

Our findings highlight a need to broaden the scope of surveillance with respect to ATP use in older adults, especially in critical illness. Providers do not currently perceive older adults to be at risk for using these products [44]. More accurately measuring ATP use in acutely ill and critically ill patients will permit better targeted interventions and directed investigation into the short-term and long-term effects of ATP use on disease. The recent outbreak of vaping-associated pulmonary illnesses demonstrates an urgent need for more accurate and meticulous ATP-exposure histories [5]. The identification of this outbreak is clear evidence that ATPs can cause severe harm to users. However, we are unable to identify more subtle health effects such as whether or not ATPs exacerbate harms related to acute or chronic illnesses because ATP use history is not commonly obtained. This is exacerbated by the fact that ATPs are extremely broad and different products will have various health effects on users. Without more accurate histories, any effects that these products may have on acute or long-term illness may be skewed or hidden by flawed measurement of product use. Furthermore, while biological measurement of acute and chronic tobacco exposure has opened an avenue for investigation of the effects of tobacco use on acute illnesses, it cannot differentiate between types of product used [35,45]. Therefore, we would advocate for a standardized assessment of ATP use in outpatient or non-acute settings so that ATP use is more completely documented in the medical record before the onset of acute illnesses.

Finally, there are challenges to fully understanding the patterns of ATP use within the acutely and critically ill. For example, we were not able to obtain survey data on ATP use from all patients, especially those who were altered or those who died. This poses a barrier for researchers who hope to investigate the acute and long-term risks associated with electronic cigarette or other ATP use in the sickest of patients. Our protocol was designed to facilitate the enrollment of critically ill patients, and our research group has a long experience in conducting clinical trials in this population [26,32,33,35,46]. First, an IRB-approved waiver of consent for minimal-risk research allowed for patients to be initially identified and enrolled but consented at a later time when the severity of their illness was improved. Patients or their surrogates had the option to complete the substance use survey at a later date, in order to respect the high-stress situations that hospitalization imposes on patients and their families. Secondly, we occasionally employed verbal witnessed consent with a nurse in order to facilitate enrollment of some patients who did not have the strength to sign the formal consent document. Thirdly, broad research staff coverage between the hours of 6 a.m. and midnight permitted more opportunities to both enroll and administer surveys to patients.

One of the strengths of this study is that it assessed the prevalence of current ATP use in a diverse, multicenter cohort of hospitalized patients with an 80% survey completion rate. This sample represents a broad and diverse population of patients from which the true prevalence of ATP use is unknown. There are several limitations to our study. First, this cohort is limited to patients recruited from one metropolitan area in one state, who may not completely reflect national trends and usage. However, the prevalence of alternative product use in our cohort is similar to published findings in PATH, NHIS, and Tobacco Products and Risk Perceptions Survey datasets, suggesting it is representative [19,40,41]. In addition, ATPs are a new area and a growing field of study, and there remains debate regarding proper terminology of products such as electronic cigarettes, which do not actually contain tobacco. However, we have elected to use ATP because this is the more commonly used abbreviation and describes non-cigarette tobacco products in the broadest sense. This terminology is used to define these products in multiple prior studies, and most government regulatory agencies refer to the products investigated in our study as ATPs. Second, within this study, we did not ask about frequency of use, specific products/brands, perceptions, or other behaviors associated with ATP use. Our survey was intentionally brief so as to limit the stress on critically ill patients and families; therefore, we were not able to fully characterize differences in usage patterns among ATP users. For example, pipe could have been interpreted as either a waterpipe or traditional European pipe, which may have explained the differences in our findings compared to PATH. We also did not specifically identify “roll your own” tobacco products, although this is certainly an increasing segment of non-cigarette product usage (up to 30% in certain populations) [47]. This may not have been captured in our survey. Thirdly, our dataset is from 2013 to 2016 and may not reflect the current patterns in ATP use. For example, ATPs now include heated tobacco products such as IQOS (brand name), which were not available in the U.S. during the study period but have since been introduced. Nevertheless, in the context of increased ATP usage in the overall population, our findings still support the need for surveillance of ATP usage in the critically ill population. Fourth, our survey was only administered once per subject, generally while the patient was in the ICU. Results may differ if respondents were approached outside of the context of a critical illness. Finally, we did not collect the relationship between the surrogate responder and the patient (e.g., spouse vs. parent vs. child vs. sibling), pre-morbid cognitive status, or measures of functional independence; all factors that could have influenced our results.

## 5. Conclusions

ATP use within critically ill patients is common and highlights an at-risk population that is not commonly studied. Development of more standardized methods for identifying ATP use will allow researchers to better determine their potential role in severe disease. As there may be an increasing prevalence of ATP use in the general population over time, further surveillance of ATP use in critically ill patients should be routinely conducted.

## Figures and Tables

**Table 1:** Demographic characteristics of the cohort.

Variable	Hospital A (*n* = 465)	Hospital B (*n* = 68)	*p*-Value	Overall (*n* = 533)
Survey Completion	368 (79)	57 (84)	0.37	425 (80)
Age, mean ± SD	66 ± 16	63 ± 13	0.32	65 ± 16
Male	257 (55)	41 (60)	0.65	298 (56)
Race			0.002 *	
African American	60 (13)	16 (24)		76 (14)
Asian	132 (28)	11 (16)		143 (27)
Caucasian	217 (47)	25 (37)		242 (45)
Other	56 (12)	16 (24)		72 (14)
Hispanic	49 (11)	15 (22)	0.01 *	64 (12)
Insurance			0.03 *	
Medicaid	92 (20)	22 (32)		114 (21)
Medicare	237 (51)	35 (51)		272 (51)
Private Insurance	128 (27)	10 (15)		134 (25)
None	10 (2)	0 (0)		10 (2)
Other	2 (0)	1 (1)		3 (1)
Cigarette Smoking History (Chart)			0.004 *	
Current Cigarette Smoker	56 (12)	19 (28)		75 (14)
Former Cigarette Smoker	163 (35)	21 (31)		184 (35)
Never Cigarette Smoker	217 (47)	23 (34)		240 (45)
Unknown	29 (6)	5 (7)		34 (6)
Cigarette Smoking History (Survey) **			0.002 *	
Current Cigarette Smoker	43 (12)	15 (26)		58 (14)
Former Cigarette Smoker	135 (37)	24 (42)		159 (38)
Never Cigarette Smoker	189 (52)	18 (32)		207 (49)
Poly-ATP Use (Survey) **	35 (10)	8 (14)	0.30	43 (10)

Note: All values are listed as *n* (%) unless otherwise specified. Categorical data were analyzed by Pearson’s chi-squared test or Fisher exact test. Continuous variables were. compared using Student’s t-test. For non-normally distributed values, Mann–Whitney U test was used. * Statistical significance was defined as *p* ≤ 0.05, using a two-tailed test of hypothesis. ** Analysis includes only subjects who completed a survey (*n* = 425).

**Table 2:** Characteristics of self-responders, alternative tobacco product (ATP) users vs. non-users.

Variable	ATP User (*n* = 44)	Non-User (*n* = 215)	*p*-Value
Hospital A vs. Hospital B			0.04 *
Hospital A	35 (80)	197 (90)	
Hospital B	9 (20)	18 (10)	
Enrolled to Hospital Floor or ICU			0.33
Hospital Floor	10 (23)	65 (30)	
ICU	34 (77)	150 (70)	
Age, mean ± SD	57 ± 17	62 ± 14	0.08
Male	36 (82)	128 (59)	0.01 *
Race			0.11
African American	7 (16)	33 (15)	
Asian	2 (5)	46 (21)	
Caucasian	28 (64)	107 (50)	
Other	7 (15)	29 (14)	
Insurance			0.19
Medicaid	12 (27)	51 (24)	
Medicare	24 (55)	101 (46)	
Private Insurance	6 (14)	61 (28)	
None	1 (2)	3 (1)	
Other	1 (2)	2 (1)	
Chronic Heart Disease	9 (20)	45 (21)	0.94
Cerebrovascular Disease	3 (7)	10 (5)	0.47
Chronic Lung Disease	12 (27)	62 (29)	0.83
Solid Tumor Malignancy	9 (20)	55 (25)	0.51
Cigarette Smoking History (Survey)			<0.001 *
Current Cigarette Smoker	18 (41)	25 (12)	
Former Cigarette Smoker	19 (43)	73 (34)	
Never Cigarette Smoker	7 (16)	116 (54)	
Alcohol Use	22 (51)	55 (27)	0.001 *
Alcohol Abuse	7 (16)	10 (5)	0.008 *
Clinical Outcomes, ICU Patients			
In-hospital Mortality	1 (3)	14 (9)	0.31
APACHE III, mean ± SD	77 ± 29	76 ± 29	0.8
Clinical Outcomes, Floor Patients			
In-hospital Mortality	1 (10)	3 (5)	0.44
APACHE III, mean ± SD	39 ± 19	47 ± 24	0.25

Note: All values are listed as *n* (%) unless otherwise specified. Categorical data were analyzed by Pearson’s chi-squared test or Fisher exact test. Continuous variables were compared using Student’s t-test. For non-normally distributed values, Mann–Whitney U test was used. * Statistical significance was defined as *p* ≤ 0.05, using a two-tailed test of hypothesis. ICU, intensive care unit; APACHE III, Acute Physiology, Age, Chronic Health Evaluation.

**Table 3:** Patient vs. surrogate reports of alternative tobacco product usage.

Variable	Self-Responder (*n* = 259)	Surrogate Responder (*n* = 166)	*p*-Value
Current ATP Use, *n* (%)	44 (17)	8 (5)	<0.001 *
Poly-ATP Use	21 (8)	2 (1)	0.01 *
Smokeless Tobacco ^++^	10 (4)	2 (1)	0.18
Electronic Cigarettes ^+++^	13 (5)	4 (2)	0.26
Cigars ^+^	23 (9)	4 (2)	0.01 *
Little Cigars ^+^	11 (4)	2 (1)	0.08
Pipe ^+^	15 (6)	2 (1)	0.04 *
Hookah ^+^	8 (3)	1 (1)	0.16

Note: All values are listed as *n* (%) unless otherwise specified. Categorical data were analyzed by Pearson’s chi-squared test or Fisher exact test. Continuous variables were compared using Student’s t-test. For non-normally distributed values, Mann–Whitney U test was used. * Statistical significance was defined as *p* ≤ 0.05, using a two-tailed test of hypothesis. ^+^ Combustible tobacco product; ^++^ non-combustible tobacco product; ^+++^ may contain nicotine or other products.

**Table 4:** Analysis of Population Assessment of Tobacco and Health (PATH), National Health Interview Survey (NHIS) and Tobacco Products and Risk Perceptions Surveys (TPRPS) cohort data ^.

Variable	EARLI (*n* = 425)	PATH (*n* = 32,320)	*p*-Value	NHIS (*n* = 36,697)	*p*-Value	TPRPS (*n* = 11,708)	*p*-Value
Electronic Cigarettes ^+++^	17 (4)	1616 (5)	0.38	1395 (3.8)	0.83	-	-
Cigars ^+^	27 (6.4)	1907 (5.9)	0.69	-	-	-	-
Cigarillos ^+^	6 (1.4)	1099 (3.4)	0.02 *	-	-	-	-
Hookah ^+^	9 (2.1)	679 (2.1)	0.98	-	-	277 (1.5)	0.31
Pipe ^+^	17 (4)	323 (1)	<0.001 *	-	-	-	-
Smokeless Tobacco ^++^	12 (2.8)	1002 (3.1)	0.74	-	-	-	-

Note: Categorical data were analyzed by Pearson’s chi-squared test or Fisher exact test. Continuous variables were compared using Student’s t-test. For non-normally distributed values, Mann–Whitney U test was used. * Statistical significance was defined as *p* ≤ 0.05, using a two-tailed test of hypothesis. ^ All values are listed as n (%) unless otherwise specified. ^+^ Combustible tobacco product; ^++^ non-combustible tobacco product; ^+++^ may contain nicotine or other products.

## Supplementary Materials

The following are available online at https://www.mdpi.com/1660-4601/17/23/8707/s1, Supplemental Figure S1. Alcohol and Cigarette Use Questionnaire; Supplemental Figure S2. Flow Diagram of Enrollment; Supplemental Table S1. Demographic Characteristics and Clinical Outcomes, Survey Responders vs. Non- Responders. APACHE: Acute Physiology and Chronic Health Evaluation; Supplemental Table S2. Demographic Characteristics, Self vs. Surrogate Responders; Supplemental Table S3. Surrogate and Self Responders Show Agreement with Medical Record on Cigarette Smoking History; Supplemental Table S4. Characteristics of Self Responders, ATP Users vs. Cigarette Smokers.
