# Supplementary material for: Alternative Tobacco Product Use in Critically Ill Patients
Source: Int J Environ Res Public Health. 2020 Nov 24;17(23):8707. doi: 10.3390/ijerph17238707 (PMC7727672; doi:10.3390/ijerph17238707)
Supplement: Supplementary file 1 [file ijerph-17-08707-s001.pdf]

Supplement

Supplemental Figure S1. Alcohol and Cigarette Use Questionnaire

Cigarette and Alcohol Questionnaire

Study ID#: ___________ **Collected by:** ___________ **Time:** ____ : _____ **Date:** ___ / ___ / ___

**Respondent:**	☐ Patient		☐ Surrogate	
**Patient's Gender:**	☐ Male		☐ Female	☐ Transgender
**Is the Patient Hispanic?**	☐ Yes		☐ No	
**Patient's Race/Ethnicity:**	☐ Caucasian/White	☐ African American/Black	☐ Pacific Islander	☐ Unknown
	☐ Asian	☐ Native American	☐ Mixed Race	☐ Other ___________

**Cigarette Smoking**	**Yes**	**No**	**Unsure**
1. Has the patient smoked more than 100 cigarettes in his/her lifetime? *(If no, skip to 5)*	☐	☐	☐
2. If yes, how many pack-years? # of packs per day ___________ * # of years smoked ___________ =	___________ pack-years		
3. Is patient currently a smoker?	☐	☐	☐
a. If yes, how many cigarettes/day?	___________ cigarettes/day		
4. If the patient is a former smoker, when did s/he quit smoking?	___________ quit date		
5. Does the patient use smokeless tobacco products? (chew, snuff)	☐	☐	☐
6. Does the patient smoke other tobacco products? *(If no, skip to 7)*	☐	☐	☐
a. ☐ Cigars ☐ Little Cigars ☐ Cigarillos ☐ Pipe ☐ Hookah ☐ Other ___________			
7. Does the patient use electronic cigarettes? (aka "vaping")	☐	☐	☐

Alcohol Use Disorder Identification Test: Interview Version

1. How often do you have a drink containing alcohol?
   (0) Never
   (1) Monthly or less
   (2) 2 to 4 times a month
   (3) 2 to 3 times a week
   (4) 4 or more times a week

   Skip to Q 9 & 10 if Q 1 = 0 ☐

2. How many drinks containing alcohol do you have on a typical day when you are drinking?
   (0) 1 or 2
   (1) 3 or 4
   (2) 5 or 6
   (3) 7, 8, or 9
   (4) 10 or more ☐

3. How often do you have six or more drinks on one occasion?
   (0) Never
   (1) Less than monthly
   (2) Monthly
   (3) Weekly
   (4) Daily or almost daily

   Skip to Q 9 & 10 if total score for Q 2 & 3 = 0 ☐

4. How often during the last year have you found that you were not able to stop drinking once you had started?
   (0) Never
   (1) Less than monthly
   (2) Monthly
   (3) Weekly
   (4) Daily or almost daily ☐

5. How often during the last year have you failed to do what was normally expected from you because of drinking?
   (0) Never
   (1) Less than monthly
   (2) Monthly
   (3) Weekly
   (4) Daily or almost daily ☐

6. How often during the last year have you needed a first drink in the morning to get yourself going after a heavy drinking session?
   (0) Never
   (1) Less than monthly
   (2) Monthly
   (3) Weekly
   (4) Daily or almost daily ☐

7. How often during the last year have you had a feeling of guilt or remorse after drinking?
   (0) Never
   (1) Less than monthly
   (2) Monthly
   (3) Weekly
   (4) Daily or almost daily ☐

8. How often during the last year have you been unable to remember what happened the night before because you had been drinking?
   (0) Never
   (1) Less than monthly
   (2) Monthly
   (3) Weekly
   (4) Daily or almost daily ☐

9. Have you or someone else been injured as a result of your drinking?
   (0) No
   (2) Yes, but not in the last year
   (4) Yes, during the last year ☐

10. Has a relative or friend or a doctor or another health worker been concerned about your drinking or suggested you cut down?
   (0) No
   (2) Yes, but not in the last year
   (4) Yes, during the last year ☐

Record total of specific items here ☐

Supplemental Figure S2. Flow Diagram of Enrollment

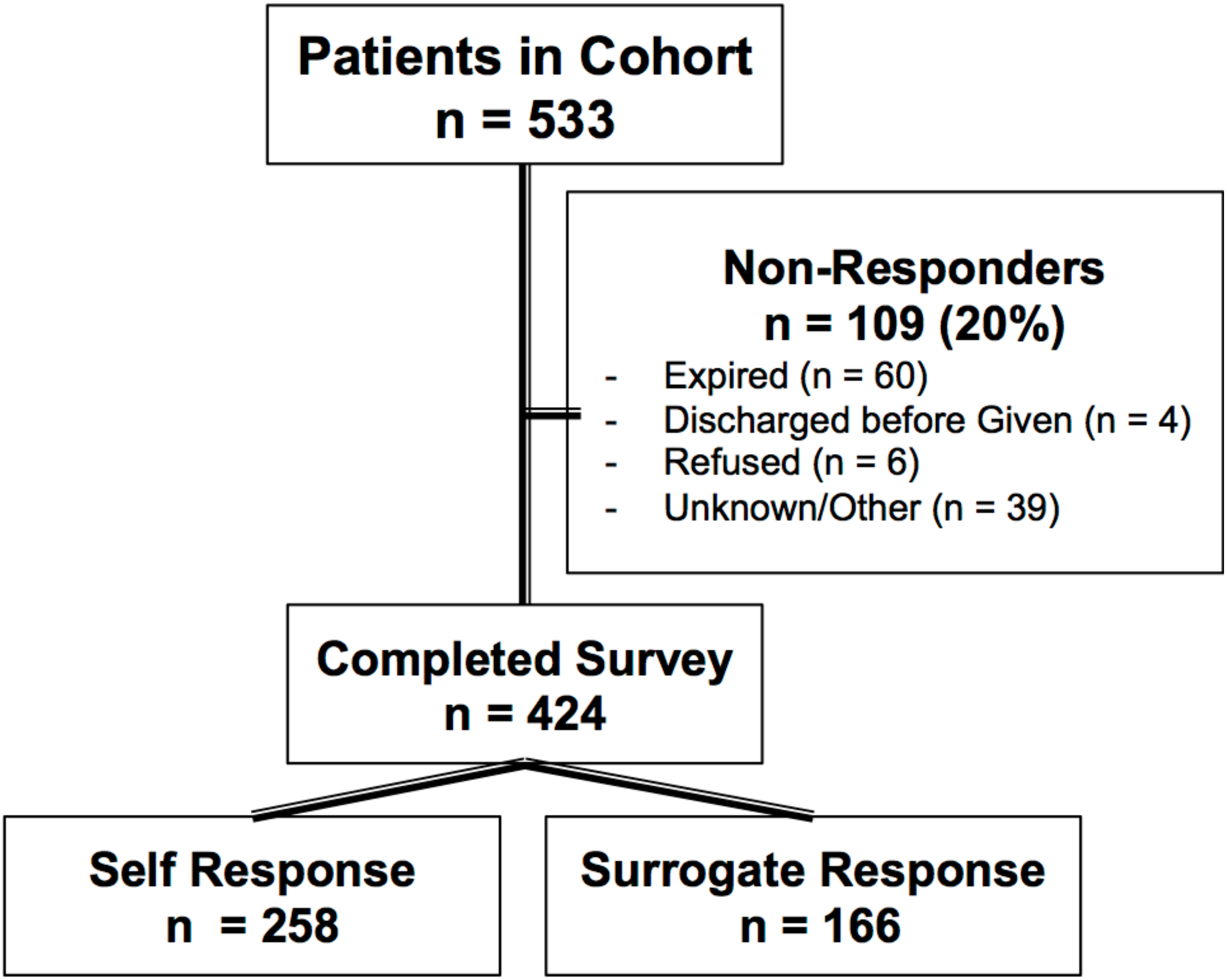

Supplemental Table S1. Demographic Characteristics and Clinical Outcomes, Survey Responders vs. Non-Responders.^

Variable	Survey Responder (n = 425)	Survey Non-Responder (n = 108)	p-value
Hospital A vs. Hospital B			0.37
Hospital A	368 (87)	97 (90)	
Hospital B	57 (13)	11 (10)	
Admitted to Floor or ICU			<0.001*
Hospital Floor	109 (26)	10 (9)	
Hospital ICU	316 (74)	98 (91)	
Age, mean ± SD	65 ± 16	69 ± 15	0.01*
Male	240 (56)	58 (54)	0.74
Race			0.94
African American	59 (14)	17 (16)	
Asian	113 (27)	30 (28)	
Caucasian	195 (46)	47 (44)	
Other	58 (14)	14 (13)	
Hispanic	51 (12)	13 (12)	0.97
Insurance			0.46
Medicaid	88 (21)	26 (23)	
Medicare	213 (50)	59 (55)	
Private Insurance	113 (27)	21 (19)	
None	7 (2)	3 (3)	
Other	3 (1)	0 (0)	
In-hospital Mortality	58 (14)	77 (71)	<0.001*
APACHEIII, mean ± SD	76 ± 35	116 ± 46	<0.001*
Primary Diagnosis (most common)			
1:	Sepsis<br>120 (22)	Sepsis<br>25 (23)	
2:	Pneumonia<br>94 (18)	Cardiac Arrest<br>20 (18)	
3:	Cardiac Arrest<br>33 (6)	Respiratory Failure<br>9 (8)	
4:	UTI<br>23 (4)	Pneumonia<br>5 (5)	

^ = all values are listed as n (%) unless otherwise specified

* Note: Categorical data were analyzed by Pearson's chi-squared test or Fischer exact test. Continuous variables were compared using Student's t-test. For non-normally distributed values, Mann-Whitney U test was used. Statistical significance was defined as $p \leq .05$, using a two-tailed test of hypothesis.

The most common diagnoses included sepsis, pneumonia, and cardiac arrest (**Supplemental Table 1**).

Supplemental Table S2. Demographic Characteristics, Self vs. Surrogate Responders

Variable	Self-Responder (n = 259)	Surrogate-Responder (n = 166)	p-value
Hospital A vs. Hospital B			0.19
Hospital A	229 (88)	141 (85)	
Hospital B	30 (12)	25 (15)	
Admitted to Floor or ICU			0.051
Hospital Floor	75 (30)	34 (22)	
Hospital ICU	184 (70)	132 (78)	
Age, median ± SD	61 ± 15	70 ± 15	<0.001*
Male	161 (62)	79 (48)	0.003*
Race			0.001*
African American	40 (15)	19 (11)	
Asian	48 (19)	65 (39)	
Caucasian	135 (52)	60 (36)	
Other	36 (14)	22 (14)	
Hispanic	32 (12)	19 (11)	0.88
From Skilled Nursing Facility/Home	32 (12)	29 (17)	0.54
Cigarette Smoking History (Chart)			0.008*
Current Smoker	49 (19)	12 (7)	
Former Smoker	88 (34)	58 (34)	
Never Smoker	118 (45)	85 (51)	
Unknown	7 (3)	13 (8)	
Cigarette Smoking History (Survey)**			0.06
Current Smoker	43 (16)	15 (9)	
Former Smoker	92 (36)	67 (40)	
Never Smoker	123 (48)	84 (51)	

^ = all values are listed as n (%) unless otherwise specified

* Note: Categorical data were analyzed by Pearson's chi-squared test or Fischer exact test. Continuous variables were compared using Student's t-test. For non-normally distributed values, Mann-Whitney U test was used. Statistical significance was defined as $p \leq .05$, using a two-tailed test of hypothesis.

** - Smoking status for 1 survey respondent was unknown

Supplemental Table S3. Surrogate and Self-Respondents Show Agreement with Medical Record on Cigarette Smoking History

	Patient-Responder			Surrogate-Responder		
Variable	Current Smoker	Former Smoker	Non Smoker	Current Smoker	Former Smoker	Non Smoker
Chart Review						
Current Smoker	35	11	2	10	1	1
Former Smoker	5	72	9	2	49	6
Non Smoker	0	7	110	2	10	72

Surrogate agreement = 85.6%; Cohen's k = 0.75;
Patient agreement = 86.1%; Cohen's k = 0.814;

Supplemental Table S4. Characteristics of Self Responders, ATP Users vs. Cigarette Smokers.^

Variable	ATP User (n = 44)	Cigarette Smoking Only (n = 25)	Non-ATP or Cigarette User (n = 188)	p-value
Hospital A vs. Hospital B				0.13
Hospital A	35 (80)	23 (92)	171 (90)	
Hospital B	9 (20)	2 (8)	19 (10)	
Enrolled to Hospital Floor or ICU				0.44
Hospital Floor	10 (23)	6 (24)	59 (32)	
ICU	34 (77)	19 (76)	124 (68)	
Age, mean ± SD	57 ± 17	58 ± 13	62 ± 14	0.07
Male	36 (82)	15 (60)	110 (58)	0.01*
Race				0.03*
African American	7 (16)	9 (36)	24 (13)	
Asian	2 (5)	2 (8)	44 (23)	
Caucasian	28 (64)	12 (48)	95 (50)	
Other	7 (15)	2 (8)	27 (14)	
Insurance				0.29
Medicaid	12 (27)	10 (40)	41 (22)	
Medicare	24 (55)	11 (44)	88 (46)	
Private Insurance	6 (14)	4 (16)	56 (29)	
None	1 (2)	0 (0)	3 (2)	
Other	1 (2)	0 (0)	2 (1)	
Cigarette Smoking History (Survey)				
Current Cigarette Smoker	18 (41)	-	-	
Former Cigarette Smoker	19 (43)	-	73 (34)	
Never Cigarette Smoker	7 (16)	-	116 (54)	
Alcohol Use	22 (51)	9 (36)	46 (25)	0.02*
Alcohol Abuse	7 (16)	3 (12)	6 (3)	0.002*
Clinical Outcomes, ICU Patients				
In-hospital Mortality	1 (3)	1 (5)	13 (10)	0.37
APACHEIII, mean ± SD	77 ± 29	74 ± 26	76 ± 29	0.79
Clinical Outcomes, Floor Patients				
In-hospital Mortality	1 (10)	0 (0)	3 (5)	0.67
APACHEIII, mean ± SD	39 ± 19	39 ± 14	48 ± 25	0.42

^ = all values are listed as n (%) unless otherwise specified

* Note: Categorical data were analyzed by Pearson's chi-squared test or Fischer exact test. Continuous variables were compared using Student's t-test. For non-normally distributed values, Mann-Whitney U test was used. Statistical significance was defined as p ≤ .05, using a two-tailed test of hypothesis.

** - Smoking status for 1 survey respondent was unknown

Main Article

Table 1. Demographic Characteristics of the Cohort. Data are presented as mean ± SD or n (%) unless otherwise indicated.
Figure 1. Prevalence of Alternative Tobacco Use. ATP: Alternative Tobacco Product.
Table 2. Demographic Characteristics of Self Responders, ATP Users vs. Non-Users.
Table 3. Patient vs. Surrogate Reports of Alternative Tobacco Product Usage.
Table 4. Analysis of PATH, NHIS, and TPPPS Cohort Data.

Supplement

Supplemental Figure S1. Alcohol and Cigarette Use Questionnaire.
Supplemental Figure S2. Flow Diagram of Enrollment.
Supplemental Table S1. Demographic Characteristics and Clinical Outcomes, Survey Responders vs. Non- Responders. APACHE: Acute Physiology and Chronic Health Evaluation.
Supplemental Table S2. Demographic Characteristics, Self vs. Surrogate Responders
Supplemental Table S3. Surrogate and Self Responders Show Agreement with Medical Record on Cigarette Smoking History.
Supplemental Table S4. Characteristics of Self Responders, ATP Users vs. Cigarette Smokers.
